# Expression and clinical significance of ITGA3 in breast cancer

**DOI:** 10.1515/med-2024-1113

**Published:** 2025-04-02

**Authors:** Huachun Song, Meihui Gao, Zheng Bao, Yong Yang, Yang Chen, Jing Hao

**Affiliations:** Department of Thyroid Breast Oncology, Yiwu Central Hospital, Yiwu, 322099, Zhejiang, China; Clinical Oncology College, Fujian Medical University, Fuzhou, 350122, Fujian, China; Operating Room, Yiwu Central Hospital, Yiwu, 322099, Zhejiang, China; Department of Pathology, Yiwu Central Hospital, Yiwu, 322099, Zhejiang, China; Department of Thyroid Breast Oncology, Yiwu Central Hospital, No. 519, Nanmen Street, Yiwu, 322099, Zhejiang, China

**Keywords:** ITGA3, breast cancer, prognosis, relapse-free survival

## Abstract

**Background:**

Integrin subunit Alpha 3 is essential for cell adhesion and movement, but its role in breast cancer (BC) is unclear. This study evaluated ITGA3 expression in BC and its clinical significance.

**Methods:**

The Human Protein Atlas (HPA), UALCAN, and Kaplan–Meier plotter were used to analyze ITGA3 in BC. ITGA3 was evaluated using receiver operating characteristic curves. The cell counting kit-8 test was used to examine the role of ITGA3 in cell proliferation.

**Results:**

BC tissues’ ITGA3 protein and mRNA levels were significantly lower than normal controls. ITGA3 was associated with better recurrence-free survival (RFS) and distant metastasis-free survival, especially in estrogen receptor (ER)-positive, Luminal B, and Luminal B subtypes. ITGA3 predicted RFS for 5 years and the response to chemotherapy. ITGA3 was associated with ER status but not age, tumor, node, metastasis stages, or tumor size. ITGA3 has a positive impact on BC cell growth.

**Conclusions:**

ITGA3 may be a predictive marker for BC and a therapeutic target. These findings need to be confirmed, and the molecular mechanisms of ITGA3 need to be clarified.

## 1 Introduction

Among cancers of the female population, breast cancer (BC) is marked by its high incidence in terms of onset and is ranked second worldwide [1,2]. At the same time, the incidence of BC is rising with a decrease in the mortality rate as an effect of active screening and early detection [3,4]. Mammary carcinoma can be a very deadly disease, and the dissemination of cancerous cells (metastasis) is widely regarded as responsible for its high fatality. One of the most striking features of BC is its heterogeneity, which extends to both surgical excision and chemotherapy.

Integrin Subunit Alpha 3 (ITGA3) is a constituent of the integrin superfamily and is a widely distributed cellular surface glycoprotein found throughout the body [5]. Adhesion molecules of the integrin family can form ligand–integrin molecules–cell skeleton transmembrane information systems after binding with their ligands, influencing cell growth, differentiation, and apoptosis [5,6]. ITGA3 overexpression has been associated with enhanced invasive and migratory capabilities in various cancer cell types. For example, reducing ITGA3 expression markedly inhibits the migration and invasion of head and neck squamous cell carcinoma (HNSCC) cells [7], the miR-199 family restrains the migration and invasion of bladder cancer cells by targeting ITGA3 [8], and miR-101 curbs the metastasis and angiogenesis of nasopharyngeal carcinoma (NPC) by targeting ITGA3 [9]. Cell microenvironment can trigger multiple signaling pathways involved in the progression of various disorders [10]. The relationship between ITGA3 and BC has garnered significant research interest. Studies indicate that ITGA3 expression levels in BC may be linked to tumor development and malignancy [11]. Previous research has found that high expression of ITGA3 may be related to the invasion and metastasis of BC [12]. However, the expression and prognosis of ITGA3 in the various subtypes of BC have not yet been reported.

## 2 Materials and methods

### 2.1 Human Protein Atlas (HPA)

HPA aims to map the entirety of human proteins within cells, tissues, and organs(www.proteinatlas.org) [13]. In this investigation, we utilized pathology atlases to demonstrate the levels of ITGA3 protein in diverse neoplasms. Furthermore, we produced immunohistochemical cartographies of ITGA3 in normal and mammary carcinoma tissue.

### 2.2 Kaplan–Meier plotter

The platform to identify and validate survival biomarkers [14,15]. To assess the predictive values of ITGA3, the cohorts were segregated into two cohorts using an automated selection process to determine the optimal cutoff. All potential cutoff values between the lower and upper quartiles were calculated, and the most effective threshold was selected as the cutoff. The two groups were then compared based on relapse-free survival, overall survival, palliative performance scale (PPS), and distant metastasis-free survival (DMFS).

### 2.3 UALCAN

UALCAN (ualcan.path.uab.edu/index.html) is a comprehensive online platform for extensively evaluating TCGA genetic expression data [16,17]. UALCAN utilizes TCGA RNA-seq data and clinical information from various cancerous forms to assess the influence.

### 2.4 Cell culture

Under saturated humidity conditions, the MDA-MB-231 and MCF-7 cell lines (ATCC, USA) were cultured in RPMI-1640 medium supplemented with 10% fetal bovine serum. The cells were maintained in a constant temperature incubator at 37°C with 5% CO_2_. Once the cells reached confluency, they were passaged.

### 2.5 Silencing and overexpression of ITGA3 using siRNA or plasmid

According to the manufacturer’s instructions, MDA-MB-231 cells were transfected with 50 nM siRNA (Life Technologies, USA) against ITGA3 using Lipofectamine2000 (Invitrogen, USA). The siRNA sequences used were as follows: 5-GUG GGA CUU AUC UGA GUA UTT-3 (sense), 5-AUA CUC AGA UAA GUC CCA CTT-3 (antisense), and si-NC, 5-UUC UCC GAA CGU GUC ACG UTT-3 (sense), 5-ACG UGA CAC GUU CGG AGA ATT-3 (antisense).

A plasmid construct for human ITGA3 was created following the previously described method [18]. MCF-7 cells were transiently transfected with the plasmid construct using Effectene (Qiagen, USA). The overexpression of ITGA3 was maintained for a minimum of 96 h, as previously reported [19]. The knockdown and overexpression of *ITGA3* were verified by reverse transcription quantitative polymerase chain reaction (RT-qPCR) assay.

### 2.6 RNA extraction and RT-qPCR assay

First, a centrifuge was pre-cooled to 4°C. Next, 75% ethanol was prepared using DEPC-treated water and stored at 4°C. A six-well plate was utilized, and 200 μl of TRIzol was added to each well to enable cell sedimentation. The cells were then resuspended by repeated pipetting and vortexing until a homogenous suspension was obtained. The suspension was then transferred to a 1.5 ml Eppendorf tube, and 40 μl of chloroform (TRIzol:chloroform = 5:1) was added and mixed thoroughly.

The samples were centrifuged at 12,000 rpm and 4°C for 10 min, resulting in a slight white precipitate at the bottom of the tube. Subsequently, 200 μl of ice-cold ethanol was added to the pellet. The pellet was then gently washed by pipetting and vortexing. The remaining white pellet was then dried by absorbing the residual liquid on the tube walls using a filter paper strip. Finally, the RNA concentration was measured by resuspending the pellet in DEPC-treated water, and the total RNA concentration was determined. The TransGen reverse transcription kit (Catalog Number: AT101-02; TransGen Biotech Co., Beijing, China) was used to synthesize cDNA. The quantitative polymerase chain reaction (qPCR) system was prepared using the TransGen qPCR kit (Catalog Number: AQ211-01; TransGen Biotech Co.). The qPCR amplification program was performed according to the instructions provided by the kit, as seen in Table 1.

**Table 1:** The reaction program of qPCR test

Temperature (°C)	Time (s)	Cycles
94	30	NA
94	5	40
60	30	40

### 2.7 Cell counting kit-8 (CCK-8) test

The CCK-8 test for MDA-MB-231 cells involves using a colorimetric method to measure cell viability and proliferation. Approximately 3,000 cells per well are seeded in a 96-well plate and treated with ITGA3 siRNA. After a specified incubation period, CCK-8 reagent was added, followed by an additional culture period. The CCK-8 reagent is reduced by viable cells to produce a yellow-colored formazan product, which is measured using a spectrophotometer (Andor Technology, DU420A-BRDD, Northern Ireland) at a wavelength of 450 nm.

### 2.8 ITGA expression by immunohistochemistry (IHC)

Thirty BC specimens diagnosed in our pathology department between April 2021 and April 2023 were included in this study. During surgery, the cancer tissue was collected, fixed in a 4% neutral methanol solution, dehydrated and paraffin-embedded, sectioned, and then placed in an oven at approximately 68°C for 20 min. The wax-melted slide was hydrated in gradient concentration ethanol, and the hydrated slide was placed in citric acid repair solution (pH = 6.0) for high-temperature and high-pressure antigen retrieval. After retrieval, the slide was rinsed thrice with phosphate-buffered saline (PBS) and blocked for 10 min by adding 8% goat serum dropwise. Following blocking, rabbit anti-human ITGA3 (Product Number: HPA008572, Sigma-Aldrich), diluted 1:100, was added and incubated overnight. The slides were then rinsed three times with PBS, and a secondary antibody was added, followed by a 20-min incubation at room temperature. After rinsing three times with PBS, 3,3′-diaminobenzidine was added for color development. Hematoxylin was used for counterstaining, and the slides were routinely dehydrated, dried after anti-blue, sealed, and stained under the microscope. PBS was chosen for the negative control, and known positive sections for the positive control.

### 2.9 IHC score

The IHC score, also known as the immunoreactive score (IRS), is a semi-quantitative method used to assess the expression of specific proteins in tissue samples through immunohistochemical staining.

The IRS is a method that combines the intensity of staining and the proportion (percentage) of cells stained positively. The staining intensity is semi-quantify as follows: 0, the expression presentation was less than gradient, and no brown particle existed; 1, dyed light yellow called weak positive (approximately right angle to horizontal); 2, stained deep sepia considered moderate positive (nearly in parallel with platitude) other stages were classified high positiveness. Please rate the percentage of positively stained cells (0–4, from 0% to >100%).

The final IRS was obtained by multiplying the staining intensity and percentage of positively stained cells to generate values ranging from 0 to 12. This score measures protein expression level, with higher scores indicating more substantial and widespread staining. The IRS categorizes samples into different expression levels: negative (0–1), weak (2–3), moderate (4–8), or firm (9–12) expression. This scoring system standardizes the interpretation of immunohistochemical results and facilitates comparisons across different studies.

### 2.10 Statistical analysis

The GraphPad software 9.0 (La Jolla, CA) was utilized to conduct all cell experiments. The mean ± standard deviation was used to express the results, and every trial was replicated three times. A *P*-value of <0.05 indicates statistical significance.


**Informed consent:** Written informed consent from all patients was obtained in any experimental work with humans.
**Ethical approval:** The current study was approved by the Ethics Committee of the Yiwu Central Hospital (YC20210411594).

## 3 Results

### 3.1 ITGA3 expression profiles in BC

**Figure 1:**
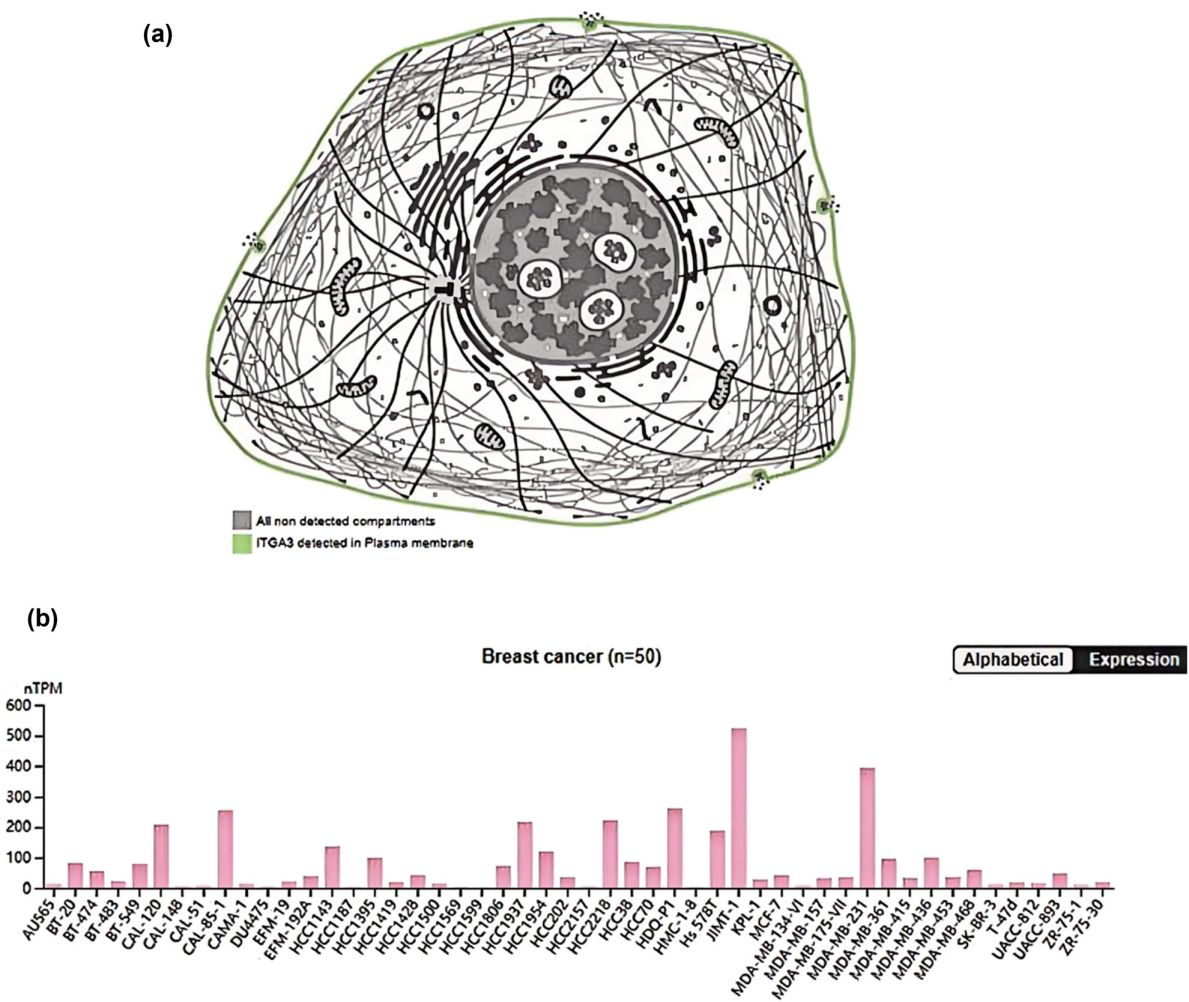
(a) The location of ITGA3 protein expressed in cells and (b) the expressed levels in the various BC cell lines (*n* = 50). nTPM: normalized transcript per million.

**Figure 2:**
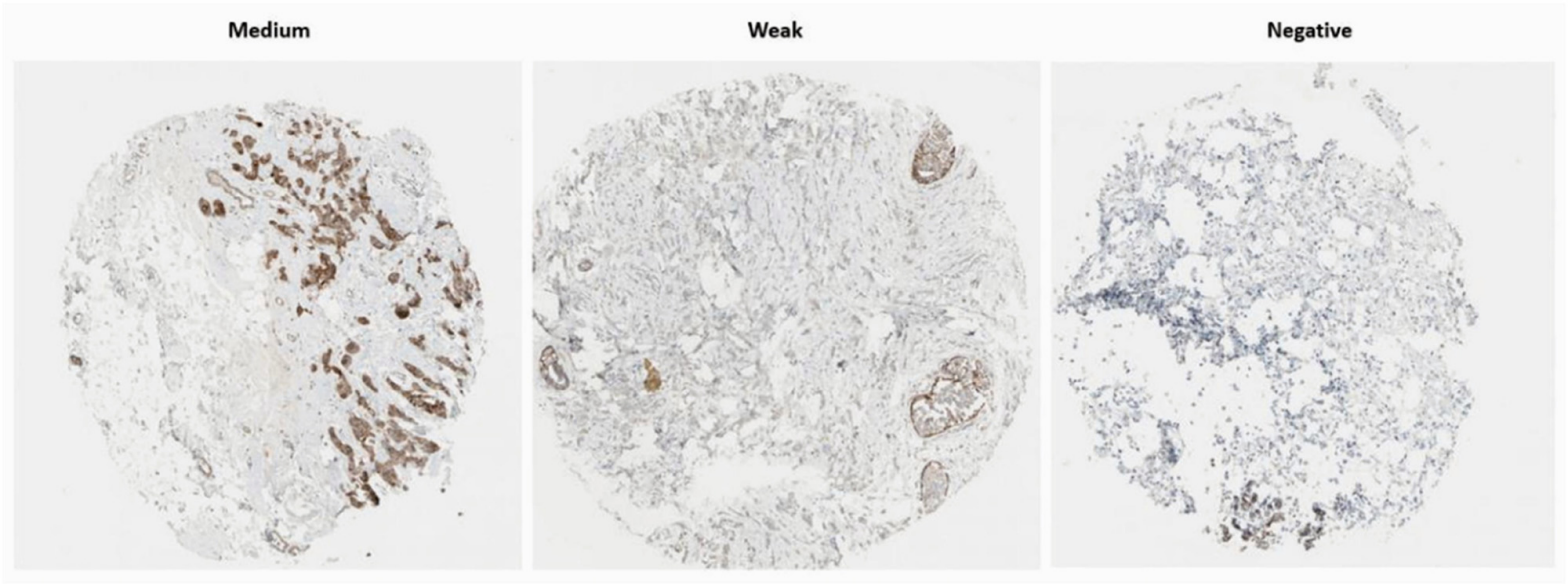
Exemplary IHC pictures portraying ITGA3 in BC tissues (scale bar = 200 mm).

The expression of ITGA3 protein was analyzed using data from the HPA database. Initial localization studies identified ITGA3 protein in the plasma membrane (Figure 1a). ITGA3 expression profiles displayed considerable heterogeneity across different BC cell lines. As illustrated in Figure 1b, ITGA3 gene expression was absent in the HCC1569 and HCC1599 cell lines, while significantly higher levels were detected in the JIMT-1 and MDA-MB-231 cell lines. Consistent with these observations, IHC images of ITGA3 across various samples revealed high heterogeneity (Figure 2).

### 3.2 ITGA3 mRNA level in different subtypes of BC

**Figure 3:**
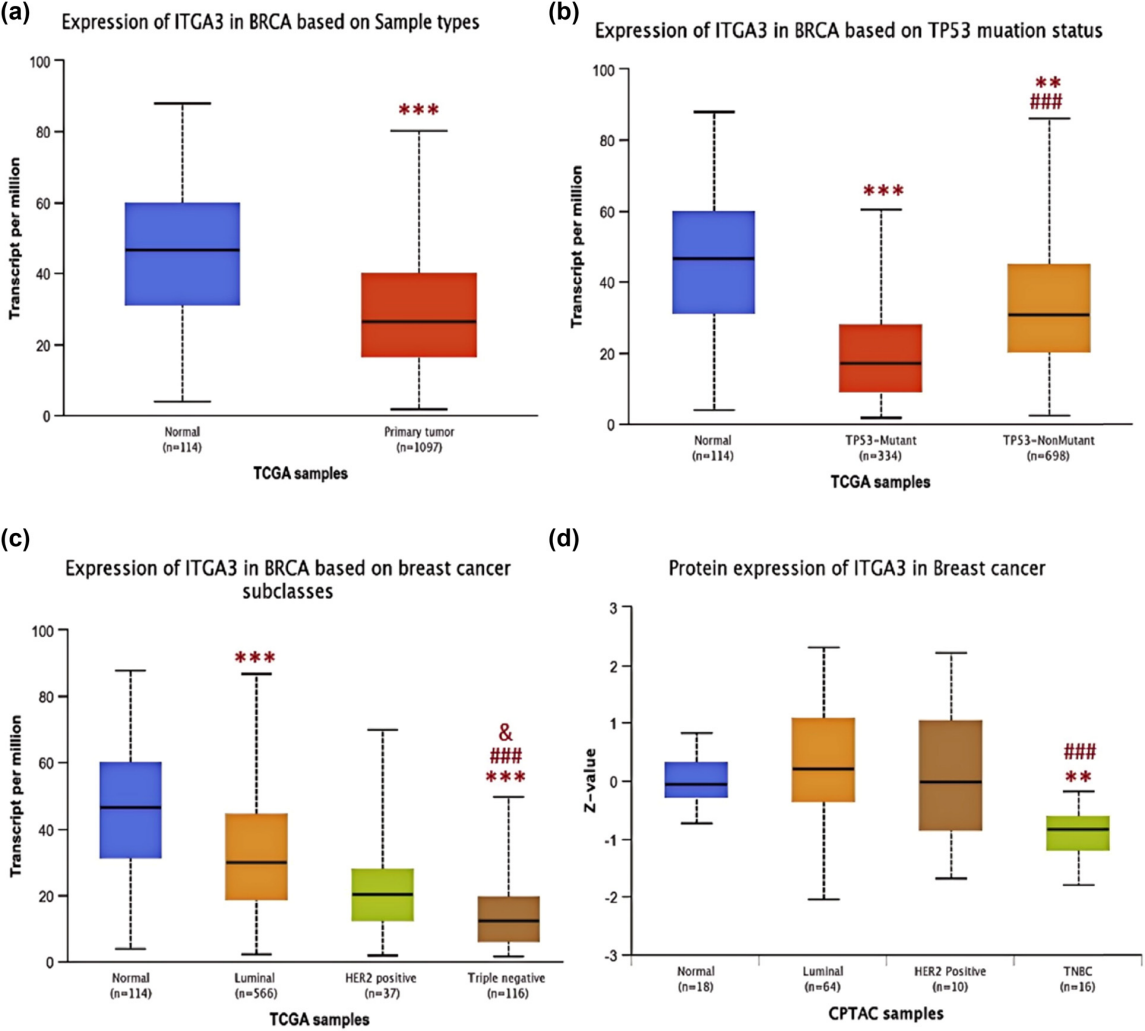
(a) ITGA3 between normal and primary BRCA groups; (b) ITGA3 in BRCA based on TP53 mutation status; (c) ITGA3 in BRCA based on subclasses; and (d) ITGA3 protein in BC in CPTAT samples.

ITGA3 expression in BC patients was markedly lower than in healthy controls (Figure 3a). In triple-negative breast cancer (TNBC) cases, ITGA3 expression was significantly lower than in the Luminal and HER2-positive subtypes (Figure 3c and d), aligning with data from CPTAC samples. Furthermore, genomic ITGA3 expression was reduced in the TP53-mutant group compared to the TP53-nonmutant group (Figure 3b).

### 3.3 Expression of ITGA3 in different histological subtypes and significant subclasses

**Figure 4:**
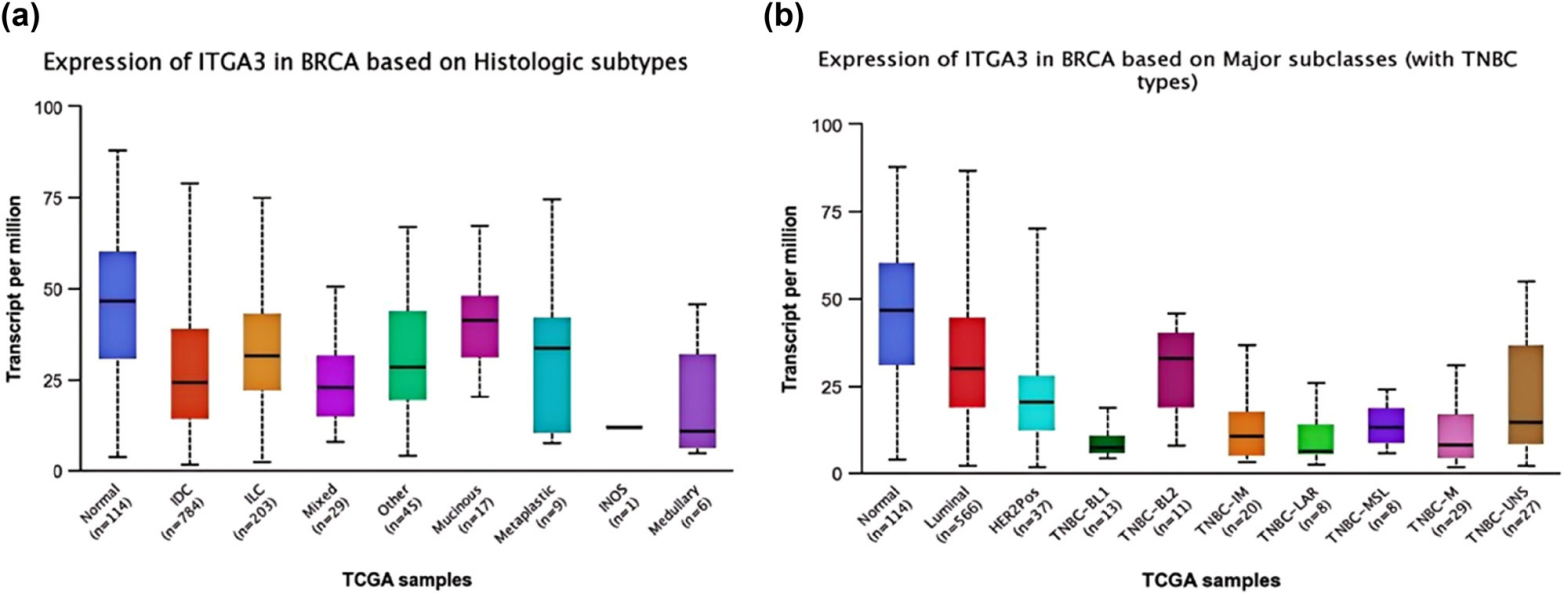
(a) ITGA3 expression in BRCA in histologic subtypes and (b) ITGA3 expression in BRCA in major subclasses with TNBC types.

The mRNA and protein expression of ITGA3 varied considerably across different histologic subtypes and major subclasses of BC (Figure 4).

### 3.4 No differences in ITGA3 mRNA levels in distinct stages and nodal metastasis status

**Figure 5:**
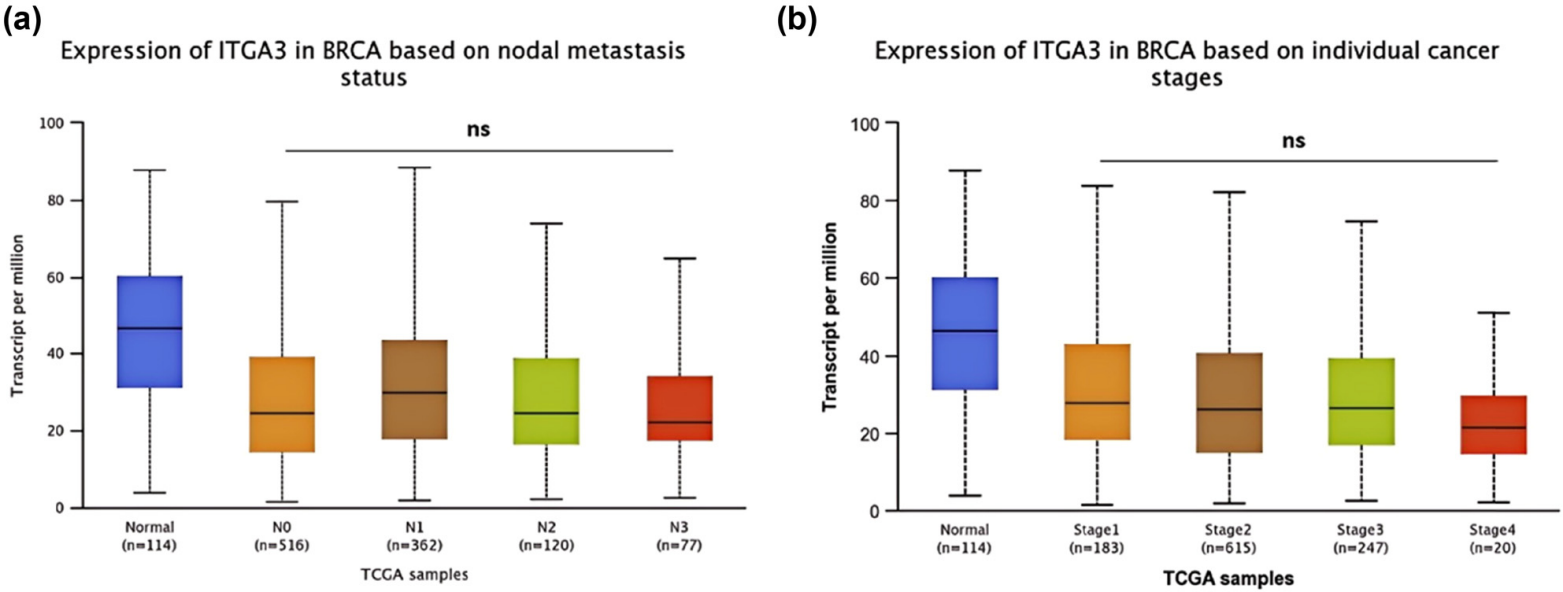
(a) ITGA3 expression in nodal metastasis status and (b) ITGA3 expression in BRCA based on individual cancer stages.

Additional detection of *ITGA3* mRNA using the UALCAN database suggested none of the differences of *ITGA3* mRNA level in distinct stages (Figure 5a) and nodal metastasis status in BRCA (Figure 5b).

### 3.5 Prognostic values of ITGA3 in BC samples

**Figure 6:**
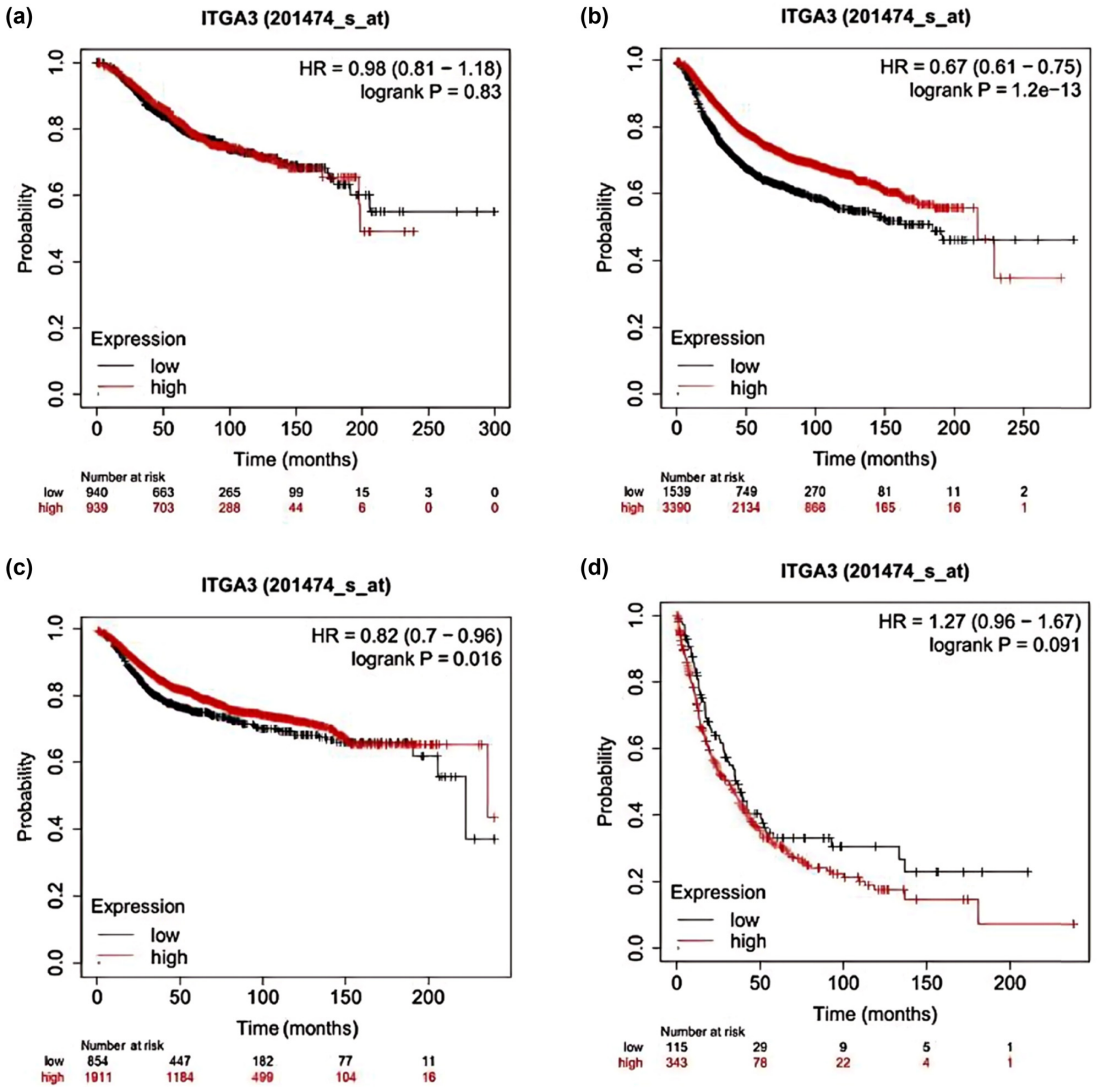
The level of ITGA3 expression is associated with prognostic outcomes. (a) OS; (b) RFS; (c) DMFS; and (d) PPS.

In contrast to Figure 6a and d, the group with higher ITGA3 expression exhibited significantly improved recurrence-free survival (RFS) (HR = 0.67 (0.61–0.75), Log-rank *P* = 1.2 × 10^−13^, Figure 6b) and DMFS (HR = 0.82 (0.7–0.96), Log-rank *P* = 0.016, Figure 6c) in BC compared to the group with lower ITGA3 expression.

**Figure 7:**
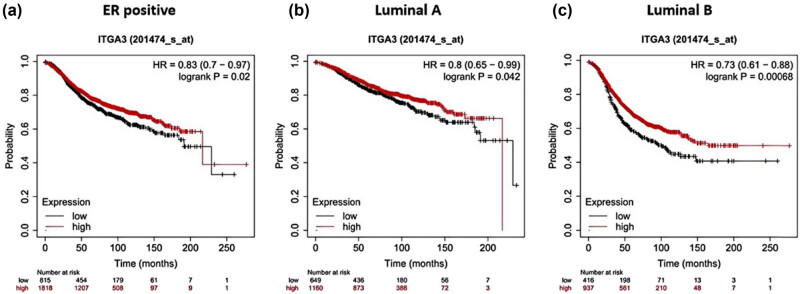
Kaplan–Meier curves of RFS in comparison between higher and lower ITGA3 expression in the different subclasses. (a) ER positive, (b) Luminal A, and (c) Luminal B.

Further analysis of ITGA3’s prognostic impact across different BC subtypes was conducted using the Kaplan–Meier plotter database. Higher ITGA3 expression was associated with significantly improved RFS in the estrogen receptor (ER)-positive cohort (HR = 0.83 (0.7–0.97), Log-rank *P* = 0.02, Figure 7a), Luminal A cohort (HR = 0.8 (0.65–0.99), Log-rank *P* = 0.042, Figure 7b), and Luminal B cohort (HR = 0.73 (0.7–0.96), Log-rank *P* = 0.00068, Figure 7c) compared to the lower expression group.

### 3.6 Response of different subtypes of BC to chemotherapy drugs

**Figure 8:**
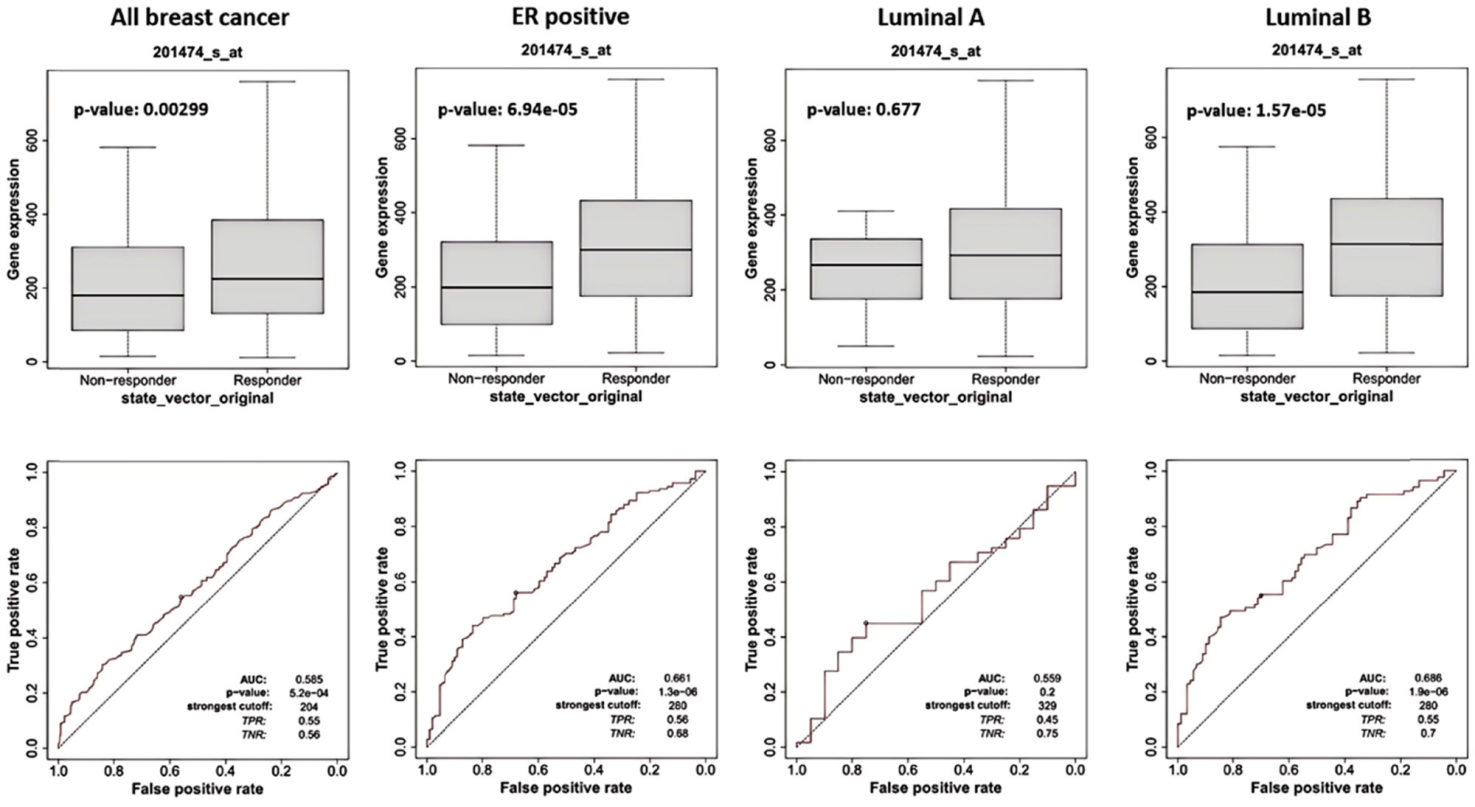
ROC curves and box-plots of ITGA3 gene in different subtypes of BC for chemotherapy drugs.

According to a previous study, we analyzed ITGA3 as a biomarker for chemotherapy in ER-positive, Luminal A, and Luminal B BC [20]. In the 5-year RFS dataset, responders were defined as patients alive 5 years after chemotherapy. Relatively higher *ITGA3* gene expression was found in the responders in ER-positive (*P* = 6.94 × 10^−5^) and Luminal B (*P* = 1.57 × 10^−5^). This suggests that chemotherapy drugs are more significantly effective for ER-positive (AUC = 0.661, *P* = 1.3 × 10^−6^) and Luminal B (AUC = 0.686, *P* = 1.9 × 10^−6^) samples. The receiver operating characteristic (ROC) plots are present in Figure 8.

### 3.7 Expression and clinical relevance of ITGA3 in BC

The patients in our study ranged from 33 to 88 years, with a mean age of 50.5 ± 11.96 years and a median age of 57 years. None had received preoperative radiotherapy or chemotherapy. Specimens were classified based on age, tumor size, tumor, node, metastasis (TNM) stage, progesterone receptor expression, and ER expression. Positive controls were derived from known positive BC tissue sections, while PBS was used instead of the primary antibody for negative controls. ITGA3 protein expression in cancerous tissues was not significantly associated with age, TNM stage, or tumor size (*P* > 0.05), but it was significantly correlated with ER expression (*P* < 0.05, Table 2).

**Table 2:** Single factor analysis affecting the prognosis of BC patients

Features	ITGA3		*χ* ^2^	*P*
	Positive (*n* = 18)	Negative (*n* = 12)		
**Age (years)**			1.8326	>0.05
≥57	12(66.7)	5(41.7)		
<57	6(33.3)	7(58.3)		
**TNM stage**			2.5000	0.1138
I/II	10(55.5)	10(83.3)		
III/IV	8(45.5)	2(16.7)		
**Tumor size (cm)**			3.2143	0.0729
Maximum diameter >2	6(33.3)	8(66.7)		
Maximum diameter ≤2	12(66.7)	4(33.3)		
**ER expression**			7.7512	0.0053
Positive	15(83.3)	4(33.3)		
Negative	3(16.7)	8(66.7)		

### 3.8 Silencing and overexpression of the ITGA3 gene can mediate the proliferative ability in mammary carcinoma cell lines

**Figure 9:**
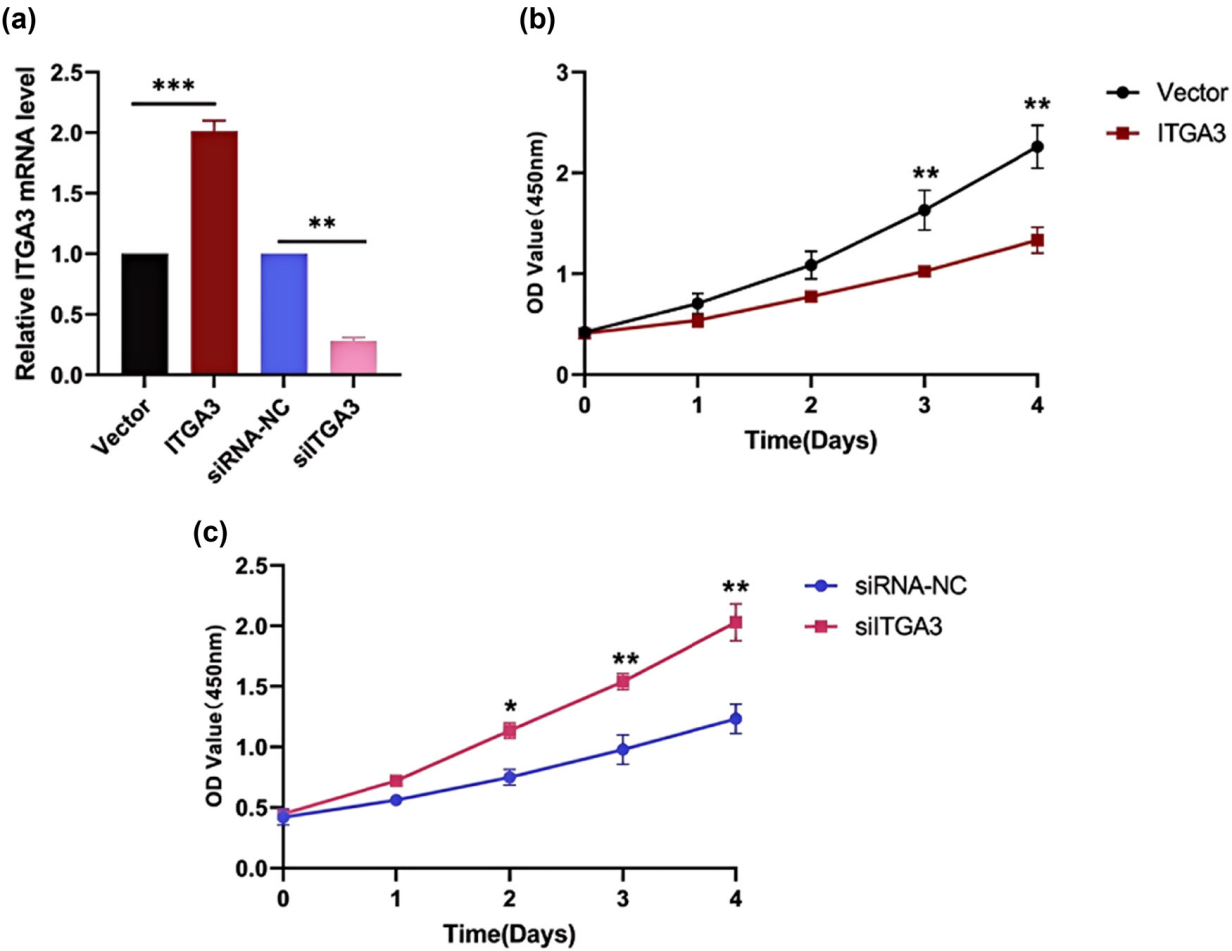
Silencing and overexpression of the ITGA3 gene can mediate the proliferative ability in cell lines of mammary carcinoma. (a) The level of the ITGA3 gene was examined by qPCR assay after transfection and transduction using siRNA and plasmid respectively. The proliferative ability was identified using the CCK-8 test in (b) MCF-7 and (c) MDA-MB-231 cells.

We selected two representative cell lines with relatively higher and lower expression of *ITGA3*: MDA-MB-231 and MCF-7, respectively (Figure 1b). The *ITGA3* mRNA levels were identified using RT-qPCR after knockdown and overexpression with siRNA and plasmid (Figure 9a). We cultured the cells under different conditions for 0–4 days (equivalent to 0–96 h) and observed changes in cell proliferation using the CCK-8 assay. The current data show that in MCF-7 cells, overexpression of the *ITGA3* gene inhibited cellular proliferation after 3 days (72 h, Figure 9b). In contrast, in MDA-MB-231 cells, silencing of the *ITGA3* gene enhanced cell proliferation after 2 days (48 h, Figure 9c).

## 4 Discussion

Recent research data indicate that mammary carcinoma, the leading type of malignant neoplasms in women, ranks second among the most common cancers worldwide [21]. Despite the tremendous progress, BC remains a significant threat to women’s health and a top priority for current biomedical research. One study found high heterogeneity of BCs in female patients younger than 45 years of age, with potentially aggressive and complex biology [22]. A study by Shirakihara et al. [23] identified ITGA3 as a potential marker for cells undergoing the epithelial–mesenchymal transition process and for invasive cancer cells. However, no study has reported the expression and clinical significance of ITGA3 in BC.

ITGA3, an integrin family member, plays an essential role in tumor cell adhesion, migration, and invasion. Research suggests that integrin signaling pathways, such as PI3K/AKT and MAPK, mediate cell proliferation and survival in cancer cells. In Luminal A and B subtypes, high ITGA3 expression may reflect a less aggressive tumor phenotype, possibly by enhancing cell adhesion to the extracellular matrix, thereby inhibiting metastatic processes. Additionally, ITGA3 may interact with ER signaling in BC, influencing the differentiation and metastatic potential of cells. We utilized multiple databases to investigate the expression, prognosis, and treatment response prediction of ITGA3 in BRCA. Our findings indicate decreased mRNA and protein levels of ITGA3 in BRCA compared to normal controls.

Furthermore, our data revealed that ITGA3 expression levels are significantly lower than healthy controls, showing apparent heterogeneity among different. Notably, the mRNA and protein expression of ITGA3 shows minimal variation across different cancer stages or nodal metastasis statuses, indicating stable expression throughout tumor progression, including tumor growth and metastasis. This stability suggests that ITGA3 may not directly contribute to the dynamic processes involved in tumor enlargement and spread. Clinically, this consistency in ITGA3 expression is significant. Given that ITGA3 levels remain primarily unaffected by tumor progression, it could serve as a dependable biomarker for prognosis, particularly within specific BC subtypes, such as ER-positive, Luminal A, and Luminal B subtypes. The uniformity of ITGA3 expression may also be advantageous in therapeutic contexts, supporting its potential as a consistent target in treatment settings. Our findings further suggest that ITGA3 could be a valuable predictive biomarker for survival outcomes in BC patients, significantly correlating with improved RFS and DMFS in the subtype mentioned above populations. Additionally, ROC analysis demonstrated that ITGA3 has substantial predictive value for 5-year RFS in ER-positive and Luminal A/B BC types. More importantly, patients with elevated ITGA3 levels exhibited a better response to chemotherapeutic drugs than those expressing low-expression ITGA3, which indicated that it could also serve as an ideal predictor for the utility of this therapeutic strategy.

Integrins are cellular membrane proteins, and ITGA3 is one such integrin [24]. The α5β1 and the related integrin complexes are transmembrane heterodimers that associate with various ligands [25]. Meanwhile, *in vitro* assays revealed that the siRNA-mediated knockdown or overexpression of ITGA3 can modulate proliferative capacity. ITGA3 was the predictive biomarker for survival in some subtypes of BRCA in our study. Overexpression of ITGA3 was significantly associated with better RFS and DMFS, especially in ER+ and Luminal A and B subtypes. This supports a model in which ITGA3 expression indicates less aggressive behaviors and better treatment response in the subtypes.

However, the role of ITGA3 appears to be somewhat context-dependent. Previous research indicates that elevated ITGA3 expression may contribute to invasion and metastasis in BC, both of which are associated with poor prognosis. The varied function of ITGA3 could be attributed to its differing biological roles across BC subtypes or stages of progression. In contrast, ITGA3 may promote cell migration and invasion in more aggressive and invasive subtypes. On the other hand, in more indolent subtypes such as Luminal A and B, higher ITGA3 expression may represent a differentiation state, thus ultimately leading to reduced metastatic capabilities. This duality emphasizes the bi-directional role of ITGA3 in BC and underscores the necessity for more investigation to define various impact mechanisms by which ITGA3 controls tumor behavior. We must utilize ITGA3 as a predictive biomarker and therapeutic target; hence, awareness of these mechanisms is crucial.

Meanwhile, ITGA3 expression in cancer tissues is investigated through several clinicopathological factors to find the between its level of gene and potential role in progressions [11,26]. In this study, we assessed the correlation of ITGA3 expression with different clinical factors such as age, TNM stage, tumor size, and ER status. ITGA3 expression was also not significantly related to age, which indicates there may be no age effect for ITGA3 in cancer tissues. This observation suggests that age might not play a significant role in regulating cancer cells for ITGA3. Second, analysis with the TNM stage did not show a significant correlation in ITGA3 expression. TNM stage is frequently taken as a sign of monitoring cancer progression and magnitude. Thus, our results did not show that the level of tumor invasion and/or regional lymph node metastasis or distant metastasis directly influenced ITGA3 expression. Thus, the result indicated that ITGA3 did not exert vital functions in tumorigenesis and metastasis-related biological processes of the TNM stage.

Furthermore, we examined the correlation between ITGA3 expression and tumor size. The results of our study demonstrated that there was no significant correlation between ITGA3 expression and the size of the tumors. The separate box represents that tumor size is typically another indicator of aggressiveness and prognosis or eventual outcomes. The finding that there is no association between ITGA3 and tumor size would indicate, to a certain extent, the economic function of the up-regulation manifestation, i.e., more significant growth impact or increasing larger masses may not be directly affected by an altering degree expression profile, due for example additional factors become persistent non-differentiated state cells contributing social properties.

High ITGA3 expression may be associated with favorable chemotherapy responses, particularly in Luminal A and B subtypes. Integrins have been implicated in enhancing chemotherapy efficacy in BC, potentially through modulating survival pathways in response to cytotoxic agents. The significant association of high ITGA3 expression with reduced recurrence risk supports its potential as a marker for treatment sensitivity in ER-positive BC patients. Further exploration into the mechanisms by which ITGA3 influences chemotherapy responses – such as its interactions with cell cycle regulators and DNA repair proteins – would help clarify its applicability as a therapeutic marker.

Oddly, an inverse correlation between ER and ITGA3 expression was found in our study. The ER is its expression is one of the most widely tested biomarkers used in BC pathology to determine treatment. Moreover, the positive correlation between ITGA3 and ER expression indicates that these two molecules may cooperate or have some common regulatory mechanisms. ITGA3 may be associated with or influenced by the epithelial apocrine pathway, frequently activated in BC development and progression. This relationship needs to be fully explored, which may enhance our current knowledge regarding the molecular dynamics of BC.

However, our study has a few limitations as well. The current study utilized a sample size of 30 cases, which, while informative for exploratory analysis, may limit the generalizability of findings due to the inherent heterogeneity of BC subtypes. Although the results provide preliminary insights into the potential role of ITGA3 as a biomarker, we acknowledge that a more extensive, multicenter dataset is necessary to validate these findings robustly. Future studies that incorporate more diverse BC subtypes, including HER2-positive and triple-negative cases, could provide a more comprehensive understanding of ITGA3’s prognostic and therapeutic relevance across varied patient populations. Second, the study was focused on ITGA3 and could disregard potential crosstalk with other molecules or pathways that may modulate its function in BC. A deeper exploration into the molecular landscape would further enrich the study. Additionally, future studies are required to define the functional interplay of ITGA3 with ER expression and how this may influence BC biology.

## 5 Conclusion

ITGA3 has the potential to serve as a predictive marker and therapeutic target for the treatment of breast carcinoma. Additional investigation is necessary to validate these discoveries and understand the underlying mechanisms through which ITGA3 functions in BC.
